# Micro-costing of dental prosthetics in jaw reconstruction

**DOI:** 10.1186/s41205-026-00333-x

**Published:** 2026-07-01

**Authors:** Aleksey D’Jamirze, Masako Dunn, Yee Mon Aung, John Martin, Dale Howes, Chris Ormsby, George A. Petrides, Rebecca L. Venchiarutti, Jonathan Clark, Timothy Manzie

**Affiliations:** 1 Department of Head and Neck Surgery, Chris O’Brien Lifehouse, 119-143 Missenden Road, Camperdown, NSW 2050 Australia; 2 Integrated Prosthetics and Reconstruction, Chris O’Brien Lifehouse Hospital, 119-143 Missenden Road, Camperdown, NSW 2050 Australia; 3 Sydney Medical School, Faculty of Medicine and Health, The University of Sydney, Anderson Stuart Building, Camperdown, NSW 2050, Australia; 4 School of Dentistry, Faculty of Medicine and Health Sciences, The University of Sydney, Sydney, NSW Australia; 5 Sydney School of Public Health, Faculty of Medicine and Health, The University of Sydney, Camperdown, NSW 2050 Australia; 6 Royal Prince Alfred Institute of Academic Surgery, Sydney Local Health District, Camperdown, NSW 2050 Australia; 7 Chris O’Brien Lifehouse Hospital, 119-143 Missenden Road, Camperdown, NSW Australia

**Keywords:** Oral cancer, Head and neck cancer, Jaw reconstruction, Dental implants, 3D resin printing, Dental prostheses, Costing

## Abstract

**Introduction:**

Dental rehabilitation has a significant impact on health-related quality of life (HRQOL) for patients undergoing microvascular reconstruction of the jaw. Virtual surgical planning (VSP) and computer-aided design and computer-aided manufacturing (CAD/CAM) technology allows for precise and timely delivery of an implant-retained dental prosthesis. This study characterizes the cost to produce a point of care (in-house) 3D-printed resin dental prostheses for patients undergoing osseous free flap reconstruction.

**Methodology:**

Implant-retained dental prostheses for patients undergoing osseous free flap reconstruction of the maxilla or mandible produced by the Integrated Prosthetics and Reconstruction (IPR) laboratory at Chris O’Brien Lifehouse Hospital between July 2023 and June 2024 were analysed. The costs of producing the prostheses were calculated using a “bottom up” approach where all activities associated with start-up, planning and fabrication were accounted for and unit costs for each activity quantified. Prostheses with incomplete data were excluded. All costs are reported in 2024 as USD.

**Results:**

Twenty-one patients met the study inclusion criteria of which the majority (*n* = 19) underwent mandibular reconstruction. Twelve patients underwent reconstruction for benign disease and 17 patients had the prosthesis placed at the primary reconstruction. The mean cost of producing the dental prosthesis was $861.72 (range: $702.78 - $1032.34). Start-up costs were the highest contributor (mean cost $333.71) per prosthesis, followed by the design/planning phase (mean cost $260.37), and fabrication phase (mean cost $267.64). The overall cost increased with increasing number of implant fixtures and the number of prosthetic units placed.

**Conclusion:**

VSP and CAD/CAM technology allows for rapid and accessible dental rehabilitation following osseous free flap reconstruction. However, the significant startup costs of these technologies are likely to be a barrier to institutions wanting to introduce point-of-care manufacturing of dental prosthetics.

## Introduction

Restoration of form and function are cardinal principles in microvascular reconstruction of the maxilla and mandible. The maxilla and mandible are integral components of the facial skeleton, contributing to speech, mastication, swallowing and aesthetics. Although it is well established that vascularized bone flaps facilitate dental rehabilitation and are associated with improved health-related quality of life (HRQOL) outcomes [1], only a minority of patients will undergo dental rehabilitation following surgery [2]. Factors that increase dental rehabilitation include the use of occlusally-driven virtual surgical planning (VSP) and the placement of endosseous dental implants at the time of primary reconstruction [3–5]. In recent years, this has been further enhanced by the placement of an immediate dental prostheses at the time of reconstruction, termed Jaw-in-a-Day^®^ [6].

The economics of surgical interventions are complex, with an intricate interplay between institutions, staffing, administration, policy and vendors [7]. As part of a health technology assessment (HTA), economic evaluation of interventions allows for inferences to be drawn pertaining to cost benefit, cost utility, and cost effectiveness to ultimately guide decision making and resource allocation [8]. However, each of these evaluation approaches rely on an accurate understanding of the true costs of the chosen technology, with micro-costing (bottom-up) remaining the most transparent approach [9]. The economics of surgical technologies and new procedures are infrequently addressed in the literature, leading to difficulties in deciding whether a health network or institution should allocate resources to introduce a new technology or intervention [10].

The manufacturing of dental prostheses incurs significant expense both from the perspective of personnel (e.g., design engineers and dental prosthetists) and infrastructure (e.g., technical and laboratory equipment) [11]. There is currently limited literature pertaining to the true costs associated with the provision of a dental prosthesis following free tissue transfer [12, 13]. Without an understanding of the attributable costs, it is not possible to quantify the value derived from the dental rehabilitation. The aim of this study was to perform an institution-specific micro-costing analysis of the design, manufacturing, and subsequent issue of 3D-printed resin dental prostheses for patients undergoing jaw reconstruction at a quaternary oncology centre without considering the costs of the surgery itself.

## Methodology

**Fig. 1:**
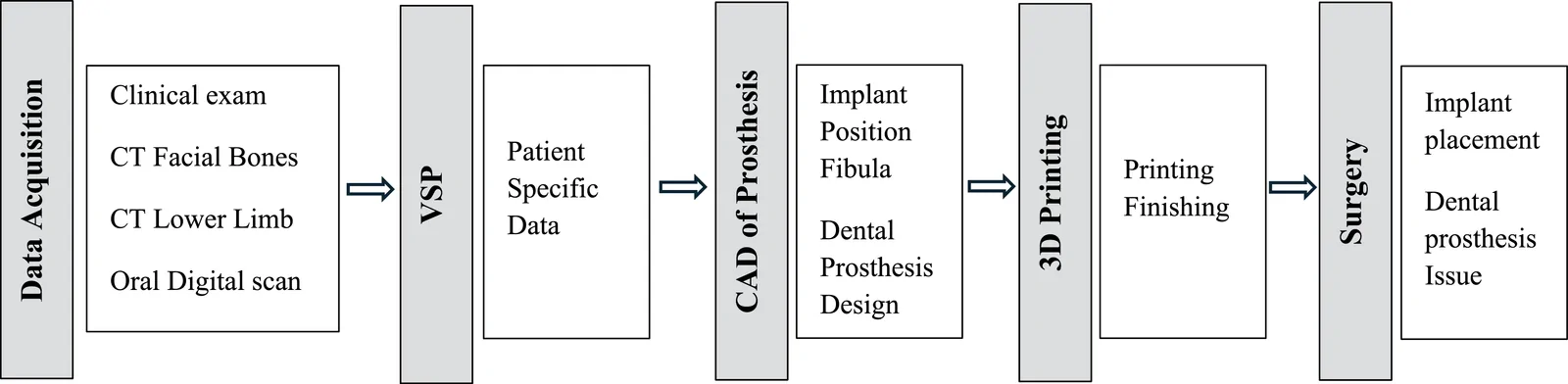
Workflow/phases for the production of the dental prosthesis

A prospective bottom-up micro-costing study from the perspective of the healthcare provider was performed for dental prosthetics produced by the Integrated Prosthetics and Reconstructive (IPR) laboratory at Chris O’Brien Lifehouse Hospital (COBLH) between July 2023 and June 2024. Patients were included if they had undergone osseous free flap reconstruction of the maxilla or mandible with placement of a dental prosthesis during their initial (primary) or a subsequent (secondary) procedure. The methodology of this micro-costing study was based on previously published guidelines and other ongoing costing projects within the institution [7, 8, 10]. All activity inputs associated with design, fabrication and preparation for issue were characterized and unit costs for each activity quantified. These activity inputs were categorized into three phases; start-up costs, planning phase, and fabrication phase and were totalled to generate an overall cost for a given prosthesis. Activities were subdivided into fixed (start-up associated) and variable costs with institution specific formulae for factors such as specialised equipment, medical devices, software, and personnel/labour. All patients underwent an identical dental prosthetic workflow (Fig. 1) for data acquisition, VSP, and the design and fabrication of the dental prosthesis. All costs were expressed in United States Dollars $USD. Costing comparisons from international literature was converted to USD based on foreign exchange rate at the time of draft publication (18/12/2024) (1.00 AUD = 0.62 USD). Ethics approval for the project was sought from local Clinical Research Ethics Committee (St Vincent’s Hospital, Sydney Australia, Human Research Ethics Committee (HREC) 2020/ETH02415).

### Start-up cost phase

The start-up costs were calculated using an amortisation model whereby systemic allocation of the depreciable amount was applied to an asset over its useful life [14]. This included equipment costs such as 3D printers, scanners, and software licenses which were used for all cases. The costs attributable to each case are demonstrated in Table 1. These costs are fixed for each case.

**Table 1:** Start-up costs associated with production of an in-house dental prosthesis

Equipment		Cost ($) USD	Purchase Year	Clinical Usage %	Cases/year	Depreciation Years	Cost/case ($USD)
Printer	Asiga Pro 4 K (Sydney, Australia)	23,505	2021	90	40	5	105.77
Software	3Shape Dental System (Copenhagen Denmark)	6,200	2023	100	100	-	12.40
Desktop 3D Scanner	Shining 3D DS-Ex Pro Scanner (Hangzhou, China)	27,280	2021	100	40	5	136.40
Intraoral 3D Scanner (IOS)	3Shape TRIOS 5 (Copenhagen Denmark)	14,193	2023	100	40	5	70.97
Prosthodontics	Prosthetic Kit (Southern Implants, South Africa)	834	2023	100	40	-	20.86

### Planning phase

The planning phase was subdivided into data preparation, treatment planning and design. Personnel involved include the surgeons, prosthodontist, biomedical engineer and dental technician. The planning phase involved a meeting with the aforementioned staff via an online platform. These sessions were conducted immediately after the surgical planning session or as a standalone session if the implants were not placed primarily.

Within this phase, costing was calculated in a cost per time value ($USD/minute) for staff involved. The salary per minute was generated for each personnel based on the COBLH Enterprise Agreements 2022 and NSW Health Award 2021 (Table 2). Logbooks were kept to record the time allocated within the planning phase, these were kept by clinical research staff and recorded for all cases undergoing virtual surgical and restorative planning. Time spent solely on implant position (if not placed primarily), abutment, prosthesis design and occlusal guide were included in calculations of VSP time for surgeons, prosthodontists, and engineers.

**Table 2:** Labour costs for personnel involved during the design and printing phase of in-house production of a dental prosthesis. (QA – Quality Assurance)

Activity		Personnel		Salary per hour ($USD)	Salary per minute ($USD)
Treatment Plan		Surgeon		133.86	2.23
		Prosthodontist		133.86	2.23
Data Preparation		Design Engineer		27.90	0.47
		Dental Prosthetist		24.92	0.42
Print Setup		Design Engineer		27.90	0.47
Post Print Processing		Dental Prosthetist		24.92	0.42
Product Check		QA Personnel		24.92	0.42

### Fabrication phase

**Fig. 2:**
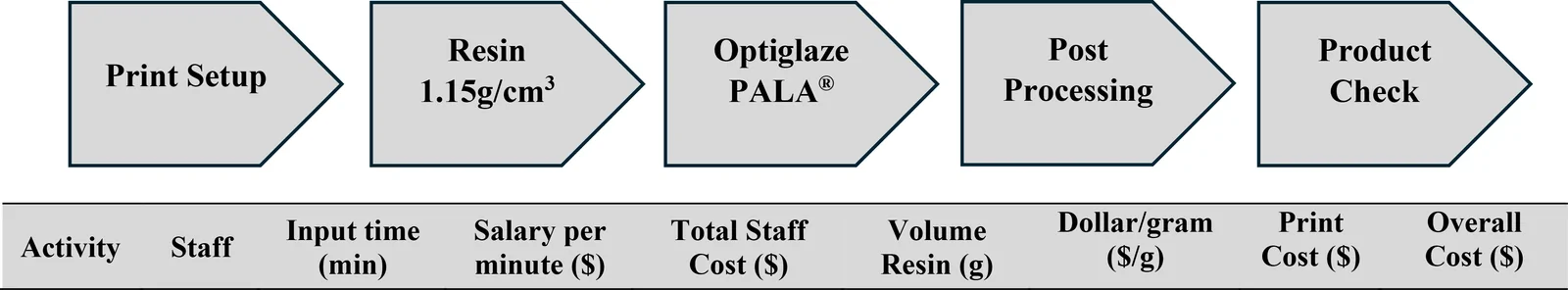
Fabrication phase workflow dental prosthesis and associated cost calculation matrix

The fabrication phase includes the labour component of engineering and laboratory personnel in addition to consumables such as implant copings, composite resin and post-processing modification (Fig. 2). The time spent by the design engineering, laboratory staff, and quality assurance personnel were calculated based on time spent on activity inputs, including print setup, additive design and post process review/modification (Table 2). Staff recorded a logbook, accurate to the nearest minute. The salary per minute data was generated as described for staff costs in a similar manner to the previous phase.

After confirmation of the surgical and dental plan, the STL file was used to 3D-printed the resin dental prosthesis. In addition to labour costs, the volume of resin and finishing products were included. Each prosthesis was printed using an Asiga Pro 4k Printer. Material costs included CROWNTEC resin (SAREMCO Dental AG. Rebstein, Switzerland) ($1.83/gram), 100% isopropyl alcohol 250 ml ($0.77/prosthesis), and Optiglaze (GC Corporation. Fuji, Japan) or PALA^®^ resin (Kulzer GmbH. Hanau, Germany) ($18.6/prosthesis). Finally, dental implant (Southern Implants, South Africa) coping costs were incorporated into the fabrication phase ($37.63 per implant fixture).

### Statistical Analysis

Costing data was analysed using Microsoft Excel (Microsoft 2024). Continuous data was reported as using mean and standard deviation. Individual data sets were assessed for normality using analytical Shapiro-Wilk testing. Inferential statistics in the form of paired t-test was carried out compare costing of prosthetics in benign and malignant disease groups with the significance threshold set at *p* < 0.05.

## Results

### Patient and prostheses characteristics

There were 21 patients who met the study inclusion criteria of whom 11 were female (52%) as summarised in Table 3. The mean age at time of prosthesis placement was 51.5 (range: 20–77 years). Twelve patients (57%) underwent treatment for benign disease. There were 61 dental implants placed into 21 fibula free flaps. The mean number of implant fixtures per case was 3 (range 1–6) and the mean number of teeth replaced was 5 (range 2–10). Seventeen patients underwent primary dental rehabilitation following a Jaw-in-a-Day^®^ protocol, and the remaining four cases underwent prosthesis placement at a subsequent surgical procedure at a mean time of 12 weeks post implant insertion. One dental implant failed to osseointegrate and was removed. All remaining implants were incorporated in the dental prostheses.

**Table 3:** Demographics of patients receiving a dental prosthesis during the period of the study at COBLH June 2023-July 2024 (CEOT - Calcifying epithelial odontogenic tumour; SCC – squamous cell carcinoma)

Patient	Age	Gender	Diagnosis	Site	Class	Reconstruction	Implants		Teeth Replaced
1	63	F	Osteoradionecrosis	Mandible	Segmental	Primary	3	5	
2	49	M	SCC	Mandible	Segmental	Primary	2	4	
3	43	M	Ameloblastoma	Mandible	Segmental	Primary	3	6	
4	55	F	Ameloblastoma	Mandible	Segmental	Primary	4	6	
5	49	F	CEOT	Maxilla	Infrastructure	Primary	3	5	
6	25	M	Ameloblastoma	Mandible	Segmental	Primary	2	4	
7	48	F	SCC	Mandible	Segmental	Primary	2	3	
8	62	F	SCC	Mandible	Segmental	Primary	2	2	
9	63	F	SCC	Mandible	Segmental	Primary	3	4	
10	33	M	Ameloblastoma	Mandible	Segmental	Primary	3	4	
11	41	F	Ameloblastoma	Mandible	Segmental	Secondary	4	6	
12	75	M	Osteosarcoma	Maxilla	Infrastructure	Primary	6	10	
13	32	M	Ameloblastoma	Mandible	Segmental	Primary	1	2	
14	74	F	Pathologic fracture	Mandible	Segmental	Primary	4	7	
15	56	F	Osteoradionecrosis	Mandible	Segmental	Primary	3	5	
16	77	M	SCC	Mandible	Segmental	Primary	2	2	
17	20	M	Ameloblastoma	Mandible	Segmental	Primary	2	2	
18	39	F	Ameloblastoma	Mandible	Segmental	Primary	3	5	
19	55	F	SCC	Mandible	Segmental	Secondary	3	6	
20	52	M	SCC	Mandible	Segmental	Secondary	3	4	
21	70	M	SCC	Mandible	Segmental	Secondary	3	5	

### Costs

The total cost of producing an in-house dental prosthesis ranged from $702.78 to $1027.38 with a mean of $861.72 (SD $81.34) per prosthesis. The cost for replacing each individual tooth ranged from $99.00 to $414.00 (mean: $205.00). Start-up costs were the highest contributor to total costs at $333.71 per prosthesis, however, this cost was the same for all cases irrespective of the number of implant fixtures used, patient demographics, or indication for surgery. The planning phase ranged from 290 min to 762 min per prosthesis (mean: 483 min) with subsequent planning costs ranging from $163.31 to $382.79 with a mean of $260.37 (SD $52.47) (Table 4). The fabrication costs ranged from $196.53 to $370.41 per prosthesis with a mean of $267.64 (SD $45.24) (Table 5). There was no difference in the total cost associated with the indication for surgery (malignancy $861.49 vs. benign $861.86, *p* = 0.49).

**Table 4:** Summary of costs for each phase of production for a dental prosthesis at COBLH (June 2023-July 2024)

Patient	Site	Gender	Age	Implants	Prosthetic Units	Starting Cost $USD	Planning Phase $USD	Fabrication Phase $USD	Total $USD
1	Mandible	F	63	3	5	333.71	382.79	310.88	1027.38
2	Mandible	M	49	2	4	333.71	245.61	290.36	869.68
3	Mandible	M	43	3	6	333.71	217.25	299.52	850.48
4	Mandible	F	55	4	6	333.71	320.01	308.54	962.26
5	Maxilla	F	49	3	5	333.71	286.53	267.39	887.64
6	Mandible	M	25	2	4	333.71	262.35	227.61	823.67
7	Mandible	F	48	2	3	333.71	231.20	239.07	803.97
8	Mandible	F	62	2	2	333.71	249.80	229.74	813.25
9	Mandible	F	63	3	4	333.71	314.43	264.11	912.25
10	Mandible	M	33	3	4	333.71	243.75	260.39	837.85
11	Mandible	F	41	4	6	333.71	214.46	257.50	805.67
12	Maxilla	M	75	6	10	333.71	289.94	370.41	994.06
13	Mandible	M	32	1	2	333.71	297.38	196.53	827.62
14	Mandible	F	74	4	7	333.71	238.17	338.77	910.66
15	Mandible	F	56	3	5	333.71	284.98	267.78	886.09
16	Mandible	M	77	2	2	333.71	172.14	196.92	702.78
17	Mandible	M	20	2	2	333.71	163.31	206.89	703.90
18	Mandible	F	39	3	5	333.71	254.45	230.91	819.07
19	Mandible	F	55	3	6	333.71	228.41	288.24	850.36
20	Mandible	M	52	3	4	333.71	237.24	292.27	863.23
21	Mandible	M	70	3	5	333.71	333.50	276.62	943.83
Mean			**51.48**	**2.90**	**4.62**	**333.71**	**260.37**	**267.64**	**861.72**

**Table 5:** Components contributing to the fabrication of prostheses issued at COBLH June 2023-July 2024

	Mean		Range	% Overall Cost (Range)
Implant Fixtures		2.9	1–6	-
Start-up Cost ($)		333.71	N/A	38.7 (32.5–47.5)
VSP/Plan Phase (min)		462.33	260–732	-
Planning Cost ($)		260.37	163.31–382.79	30.2 (23.2–37.3)
Resin Volume (cm^3^)		30.88	15.63–50.05	-
Fabrication Cost ($)		267.64	196.53–370.41	31.1 (23.8–37.2)
Total Cost ($)		861.72	702.78–1,027.38	100

## Discussion

Dental rehabilitation is important for improving functional and psychosocial outcomes after jaw reconstruction (1). There is growing interest in making dental rehabilitation more accessible and timelier [15–17]. Due to limitations in government and insurance funding, the cost of dental prosthetics can be a major barrier to uptake. Technological advancements, such as the use of occlusal-based VSP, intraoral scanning, and 3D-printing allows for a complete digital workflow that has the potential to improve efficiency through better planning and improve accessibility by reducing reliance on traditional prosthetic design and manufacturing expertise. However, some technologies may make the process more costly compared with traditional analogue techniques [18].

There is limited literature regarding the costs of producing an in-house dental prosthesis. Our study found that the mean cost was $USD 861.72 per dental prosthesis. In comparison, Williams et al. describe a series of 12 patients undergoing Jaw-in-a-Day surgery where costs were compared between commercial laboratory versus in-house produced dental prostheses. In that study, the prostheses produced by a commercial laboratory were significantly more expensive $USD 617 versus $USD 8.34 for in-house production in addition to a delay of approximately 2 weeks for fabrication [6, 13]. Their comparison underestimates the cost of in-house production by describing exclusively the printing costs. However significant start up and planning costs are also required in order for in-house printing of a prosthesis to be possible. Further, the software used in that study is not regulated for use in Australia for the production of dental prostheses and thus is not available to this institution. Sukato et al. compared in-house CAD/CAM workflow for prostheses with that of laboratory-based design and fabrication noting increased temporal and financial burden of the latter, however, did not include specific costing analysis of either process [12]. Comparatively, Chang et al. and colleagues reported individual case costs of between $USD 2,790 – $9,151 per case [18]. These costs included material and personnel costs for ablative and reconstruction planning and costs for fabricating custom reconstructive plates as well as the dental prosthesis. It is likely that surgical planning costs contributed substantially to the total costs; unfortunately, the isolated costs of the dental prosthesis were not provided [18].

In our study, start-up costs were the major contributor, contributing 39% of the total cost. While mentioned in other studies, the start-up phase has not been quantified, which grossly under-estimates the cost of in-house production [12]. Given these items have ongoing expense through maintenance, consumables and eventual replacement costs, an amortization model best demonstrates its contribution to overall costs [14]. When scaled, these start-up costs decrease considerably as a proportion of total cost, however additional personnel and labour is required which is captured in the design and fabrication phases in the data presented. In addition to the economy of scale phenomenon, lower cost printers and scanners may offer an equivalent quality while reducing the initial costs for institutions establishing an inhouse service.

The planning phase costs incurred were primarily comprised of labour costs. Logbooks were kept to accurately reflect the time spent by all involved personnel. Within this cohort of patients, this phase demonstrated the highest variability ranging from 290 min to 762 min, which accounts for the variability in total costs between individual cases. An important determinant of planning time was whether the patient had pre-existing teeth within the planned defect which could be used for the occlusal design. One would anticipate that the associated labour times will lessen with increased experience, we were unable to demonstrate this in our small cohort.

With regard to fabrication phase, the time required for post-processing (removal of printing supports, addition of resin and polishing) demonstrated little variability. Williams et al. in 2020 compared resin costs for printed prostheses at $USD 3.03–25.3 per case compared with $USD 490–1,295 for outside laboratory fabrication [13]. In contrast, in our cohort the resin costs were substantially higher ($37.78 - $103.86 per prosthesis). This difference is likely explained by variability in currency exchange, local product availability, and the requirement to use resin that is approved by the Therapeutic Goods Administration (local equivalent of the FDA) limiting lower-cost options. In addition, it is unclear how the implant components (metal copings and prosthetic screws), which are necessary in any prosthesis, were accounted for in previous published studies. The high implant utilisation (all integrated implants were included in the prosthesis) reflects the ability to attain primary stability and a standardised approach to planning the most distal implant. This implant location is a key determinant of utilisation due to the anticipated effects of trismus. Thus, we do not extend the dental prosthesis behind a first molar position which allows for easier cleaning and access by the restoring practitioner and patient [19, 20].

## Conclusion

Early dental rehabilitation has become more common in head and neck reconstruction. This micro-costing analysis provides a detailed breakdown of the start-up, design, and fabrication costs (including associated labour costs) for production of a point-of-care manufactured dental prosthesis. In-house design and fabrication of dental prostheses is likely advantageous from a cost perspective, however, this requires significant initial expenditure and staffing allocation, expertise and resources.

## Data Availability

The datasets used and/or analysed during the current study are available from the corresponding author on reasonable request.
